# Detection of Saffron’s Main Bioactive Compounds and Their Relationship with Commercial Quality

**DOI:** 10.3390/foods11203245

**Published:** 2022-10-18

**Authors:** Raul Avila-Sosa, Guadalupe Virginia Nevárez-Moorillón, Carlos Enrique Ochoa-Velasco, Addí Rhode Navarro-Cruz, Paola Hernández-Carranza, Teresa Soledad Cid-Pérez

**Affiliations:** 1 Facultad de Ciencias Químicas, Benemérita Universidad Autónoma de Puebla, Edificio 105E, 14 Sur y Av. San Claudio, Ciudad Universitaria, Col. San Manuel, Puebla 72420, Mexico; 2 Facultad de Ciencias Químicas, Universidad Autónoma de Chihuahua, Circuito Universitario s/n Campus, Universitario II, Chihuahua 31125, Mexico

**Keywords:** saffron, bioactive compounds, chemometric methods, adulteration, by-products

## Abstract

This review aims to evaluate the state of saffron’s main bioactive compounds and their relationship with its commercial quality. Saffron is the commercial name for the dried red stigmas of the *Crocus sativus* L. flower. It owes its sensory and functional properties mainly to the presence of its carotenoid derivatives, synthesized throughout flowering and also during the whole production process. These compounds include crocin, crocetin, picrocrocin, and safranal, which are bioactive metabolites. Saffron’s commercial value is determined according to the ISO/TS3632 standard that determines their main apocatotenoids. Other techniques such as chromatography (gas and liquid) are used to detect the apocarotenoids. This, together with the determination of spectral fingerprinting or chemo typing are essential for saffron identification. The determination of the specific chemical markers coupled with chemometric methods favors the discrimination of adulterated samples, possible plants, or adulterating compounds and even the concentrations at which these are obtained. Chemical characterization and concentration of various compounds could be affected by saffron’s geographical origin and harvest/postharvest characteristics. The large number of chemical compounds found in the by-products (flower parts) of saffron (catechin, quercetin, delphinidin, etc.) make it an interesting aromatic spice as a colorant, antioxidant, and source of phytochemicals, which can also bring additional economic value to the most expensive aromatic species in the world.

## 1. Introduction

Plants and vegetables are major sources of food bioactives. Spices and herbs are plant materials that provide a wide range of biologically active compounds. In addition to being used as sources of aroma, flavor, and color and as preservatives, spices and herbs have been used for medicinal purposes and health and wellness for centuries. Aromatic spices can be added to food in their natural state as a powder or extract [1]. In the food industry, it is not only the active parts of vegetables or plants that are important since there are several uses for their waste or by-products as ingredients in different food formulations [2].

Saffron is the commercial name for the dried red stigmas of the *Crocus sativus* L. flower. It is appreciated for adding color, flavor, and a particular aroma to different food dishes or drinks (paella in Spain, Milanese risotto in Italy, lussekatter buns in Sweden, and alcoholic beverages). It is considered a high-priced condiment (1500–2200 euro/kg) due to the considerable labor involved in its production since it requires manual harvesting as well as a laborious handling process (sorting, drying, and storage) [3,4,5,6]. Saffron’s principal producers are Iran and Spain, whereas the leading importers are Spain, Hong Kong, and the United States [3]. Saffron’s quality is essential for consumers in the food industry [7] and is based on the concentration of its apocarotenoids and their respective sensory attributes: crocin’s coloring strength, picrocrocin’s bitter taste, and safranal’s aromatic intensity. Saffron contains over 150 volatile and non-volatile compounds including proteins, carbohydrates, vitamins, amino acids, minerals, gums, and other compounds [8,9]. However, the apocarotenoids (crocin, picrocrocin, and safranal) are responsible for saffron’s sensorial attributes and are the major bioactive compounds used as markers for its quality. Furthermore, the quality and, consequently, the commercial value of saffron are based on the estimation of its coloring power, bitter taste, and aroma [10].

Reductions in saffron’s commercial quality can be attributed to inappropriate harvesting methods, insufficient dehydration processing, exposure to direct sunlight, improper storage, and adulteration [4,5]. Saffron fraud is related to unfair competition, including (a) by adding substances (parts of other, cheaper plants or synthetic dyes) to produce low-cost spices [5,7,11] or (b) spices that carry the Protected Designations of Origin (PDO) logo without being produced or processed in the specified geographic area [11,12]. To prevent adulteration, it is necessary to establish a precise chemical identification protocol to protect producers’ and consumers’ interests [13]. Metabolic and chemical profiling is a valuable tool for product standardization and for detecting mislabeled or fraudulent samples [4]. This review aims to evaluate the state of saffron’s main bioactive compounds and their relationship with its commercial quality. To fully achieve this purpose, the following topics are addressed: (i) we describe the *C. sativus* characteristics for obtaining saffron and its uses in the food industry; (ii) we present and discuss saffron’s chemical composition, its main bioactive compounds, and their determinations; (iii) we explain saffron’s quality compounds related to color, odor, and flavor; (iv) we differentiate the saffron authentication techniques and their relationships with chemical compounds and chemometric methods as a critical parameter of its commercial quality; and (v) we consider the saffron by-products and their applications in the food industry.

## 2. *C. sativus*

*C. sativus* belongs to the Iridaceae family and is considered a sterile herb from the *Crocus* genus [14,15,16,17,18]. It is a perennial plant; therefore, soil fertility must be carefully controlled to achieve high production. Its cultivation is adapted to arid and semi-arid lands. It grows abundantly in regions with cold winters and abundant rain in spring and autumn and low rainfall in summer; it can also grow in temperate and subtropical climates with sandy or clay soils with good drainage. Saffron is grown in Iran, Spain, India, and Greece. The plant is small, with a height of up to 30 cm. Predominantly, it consists of leaves (deep green), flowers, and a globular underground corm or bulb measuring 3 to 5 cm in diameter [14,19,20,21]. The flowers are composed of six tepals; inside the flower, three stamens are present, and a filiform white style terminates in a stigma divided into three threads. During its development and growth, the stigma changes color from white to scarlet [16,18,22,23,24,25,26]. The stigma constitutes between 7 and 7.4% of the flower and the remaining 93% is composed of the petals, stamens, and style. Stigmas represent the unique, marketable part; the rest of the plant is called the floral biomass [14]. Saffron flowers are sterile; therefore, they do not produce viable seeds and must be propagated manually by planting corms that grow underground. Flowering occurs approximately 40 days after sowing and lasts from 20 to 30 days [16,17,18,25,26]. Corms remain dormant during summer and grow at the end of the season [16,17]. The geographical origin and their respective environmental conditions (altitude, temperature, rainfall, irrigation cycles, harvest season, humidity, and properties or type of soil) influence plant growth and development, exerting strong effects on the production of secondary metabolites [6,27].

## 3. From *C. sativus* to Saffron

There are a variety of methodologies and techniques for obtaining saffron from the *C. sativus* flower. The main phases are described in the following subsections. 

### 3.1. Harvesting

Harvesting begins in the morning (deep red stigma); flowers are cut before the tepals open to prevent them from wilting in the sun (causing loss of color and concentration of apocarotenoids). This break is made in the lower part of the corolla. Fresh-cut flowers should be kept in good storage conditions, with high humidity, a low temperature, and moderate airflow. This is due to their short shelf life, rapid senescence, high water loss, and high likelihood of contamination by bacteria and fungi [3,28,29].

### 3.2. Post-Harvest

Cut flowers are transferred in baskets or sacks to the processing area, avoiding pressure or deformation of the stigmas (in Greece, stigmas are cut on the plant). Next, they are placed on a table for “monda” (separation of tepals and removal of the styles). The flower is opened and the stigmas are separated from the tepals and stamens (the stigma is cut at the base of the filaments and the style is removed). The whole, manually performed operation takes around 4 s per flower (a step responsible for the high cost). The stigmas are collected manually to preserve the bioactive compounds. However, easy degradation in the presence of light or oxidizing agents means that few stigmas are classified as high-quality saffron. Poor hygiene, transportation, bulk storage, manual harvesting, monda, sudden rain during flowering, and prolonged and inadequate storage temperatures are critical factors of quality and contamination. Accelerated stigma separation after flower harvesting is recommended to reduce these factors [3,17,28,29,30,31].

### 3.3. Drying

As described above, fresh stigmas do not transmit the typical color, flavor, and aroma so a drying treatment is necessary. This step is crucial and essential to convert *C. sativus* stigmas into the aromatic spice saffron. In most cases, it can be stated that the drying method affects the color, morphological characteristics, bioactive composition, flavor, and aroma of saffron; this is explained by the fact that, during the process, a series of biochemical and enzymatic changes occur, generating volatile and non-volatile compounds. There are several techniques for carrying out the drying process (conventional: room or moderate temperatures of 35–45 °C over long periods of time are recommended; non-conventional: very short periods of time at high temperatures of 60–70 °C) and each method has its own variables (place, temperature, relative humidity, raw material load, etc.). The variables differ between countries and according to the experience, available resources, and climate of each region, which results in variations in saffron quality [32,33,34,35,36].

### 3.4. Storage

Dried stigmas are packed in sealed containers away from moisture and light at temperatures between 5 and 25 °C. Saffron is marketed as strands or ground saffron. One kilogram of dry saffron requires between 110,000 and 165,000 flowers, which implies around 50 h of labor to pick the flowers plus 200 h to peel the stigmas from them. Storage favors the oxidative and hydrolytic decomposition of the secondary metabolites (crocin and picrocrocin). However, inadequate storage can affect the properties of the finished product [30,36,37]. 

## 4. Saffron in the Food Industry

Saffron’s aroma develops during the drying and storage stages. However, the loss of apocarotenoid quality occurs due to poor harvesting, inadequate drying and storage conditions, the mixing of stigmas with other parts of the plant, etc. [10,36,38]. It is traditionally used in industry as a medicine, textile dye [8], cosmetic raw material, ornamental flower [35], and aphrodisiac [39]. Specifically, it is used in the food industry and cuisines worldwide as a spice or seasoning [8,40], acting as a flavoring and coloring agent [39,41,42,43]. However, it is also recognized as a medicinal plant [41], carrying various beneficial health properties such as analgesic, sedative, antioxidant, anticancer, and other therapeutic properties [39]. 

Saffron has been used in the food industry for culinary purposes as an aromatic, flavoring, and coloring agent, in many products. The chemical composition of saffron makes it a valuable functional ingredient for various products in the food industry [14]. Saffron has been added to several formulations for the development of functional foods as a preservative, colorant, flavoring, antioxidant, base for bioactive compounds, etc.

Regarding the bakery and confectionery industries, Gani et al. (2021) produced fortified cookies with encapsulated bioactive compounds from saffron. The additions enhanced its antioxidant activity, providing a better color and suitable stability. In addition, in vitro digestibility showed a low glycemic index [44]. Moreover, Bhat et al. (2018) designed whole-wheat flour cookies with saffron extracts. They reported acceptable sensory characteristics (except texture), antioxidant properties, and a suitable shelf life [45]. Bhat et al. (2022) produced functional cakes from whole-wheat flour combined with saffron or tomato extracts. The addition of saffron extract to the cakes produced desserts with improved antioxidant properties, without affecting the product’s sensory quality [46]. Armellini et al. (2018) evaluated the qualities (texture, physicochemical, and sensory) of dough enriched with saffron powder. The results showed that saffron provided better textural properties, higher sensory acceptability (visual appearance, color, aroma, flavor, chewiness, hardness, gumminess, and overall acceptability), and improved antioxidant activity (higher values of crocin) in the saffron-enriched dough [47]. The same research group [43] studied the effect of saffron extract addition on starch digestibility and crocin fate and release at different cooking times in fresh pasta. The results showed that the saffron extract affected the digestibility and glycemic index. The higher the saffron concentration and the shorter the cooking time, the higher the amount of crocin released in the digestive fluids.

Sena-Moreno et al. (2018) used a saffron extract (rich in safranal) as a flavoring agent in olive oil. They reported that small concentrations of safranal led to organoleptic improvements in the oils. In addition, positive values were obtained for oxidative stability, indicating this product’s potential in the charcuterie market [48]. Almodóvar et al. (2018) compared the advantages and culinary applications of a natural commercial saffron extract (affron^®^eye) vs. saffron stigmas in refrigerated foods. They demonstrated that affron^®^eye has advantages in terms of microbiological safety, ease of dissolution, quick application, and simple mixing of ingredients [49]. Finally, Moghaddam et al. (2018) developed a probiotic beverage (*Lactobacillus*, *Lactococcus*) fermented using saffron petals. They reported its physicochemical, antioxidant, rheological, and sensory properties, showing overall benefits in terms of antioxidant and phenolic activity after fermentation [50].

## 5. Saffron’s Chemical Composition

Saffron contains more than 150 compounds (volatile and non-volatile) including carotenoids (crocetin, crocin, β-carotene, lycopene, and zeaxanthin), monoterpene aldehydes (picrocrocin and safranal), monoterpenoids, and isopherones [8,28]. However, it also contains other compounds such as flavonoids, vitamins, proteins, and amino acids [51]. Saffron owes its sensory and functional properties mainly to the presence of its carotenoid derivatives, synthesized throughout flowering but also during the whole production process [43]. These compounds include crocin, crocetin, picrocrocin, and safranal, which are the secondary or bioactive metabolites [8,43,44,52]. Saffron’s quality depends on its chemical profile and is directly related to the geographic area, climate variability, environmental practices, genetic traits, soil composition, cultivation conditions, and processing and storage methods [53,54]. Nevertheless, according to the ISO standards (3632-1:2011 and ISO 3632-2:2010), the value and quality of the stigma are measured based on the content of the color components (crocin and crocetin), the bitter taste component (picrocrocin), and the volatile compounds responsible for the odor and aroma (safranal). These specific parameters are influenced by the environmental conditions, extraction method, purification, etc. [14,28,55,56,57]. Some studies have been conducted on the extraction of bioactive compounds from saffron using the concept of green chemistry [58]. Some research on saffron stability demonstrates that temperature and humidity exert a strong influence on the degradation of the principal active ingredients [8].

### 5.1. Saffron’s Important Apocarotenoids

Crocin: The main bioactive compound of saffron was isolated by Aschoff in 1818, reporting a family of yellowish-red water-soluble carotenoids (mono-glycosyl or di-glycosyl-polyene esters) of 20 carbons [8,34,58,59,60]. In other words, this was a group of compounds formed by crocetin esterification (dicarboxylic carotenoid), which were classified according to their sugar fractions [59]. The abbreviations used in this review are as follows. The cis/trans-X-R1R2 crocin abbreviation system is used based on three main characteristics: (a) cis/trans isomers, (b) X: number of glucose components (1–5), and (c) type of structure in R1 and R2 (acid form: H; glucose: g; gentiobiose: G; Neapolitan: n; or triglucose: t.) (Suchareau et al. (2021)). The most represented crocins are trans-4-GG, trans-3-Gg, trans-2-G, trans-2-gg, trans-5-tG, and trans-1-g, among others [19,59,61,62,63,64,65,66,67,68,69,70]. 

Crocins are unusual apocarotenoids since their terminal glycoside rings confer high solubility. These pigments are detected in the red lobes of the stigmas of the *Crocus sativus* flower [14,19] and their content is proportional to the color and quality index. However, it should be noted that zeaxanthin (fat-soluble carotenoid) can also influence the color [35]. Crocins as such have low stability and lose their functionality during exposure to heat, oxygen, light absorption, acidic environments, and/or due to the presence of additives [43]. Therefore, the drying and storage temperatures are important for proper color development [68]; poor storage conditions lead to color pigment degradation [71]. Several factors are related to the concentration of these pigments in saffron stigmas, which are mainly the geographical growing region, crop conditions, type of soil, plant genetic traits, climate, planting time (rate), seed/crown rate, planting depth, corm size/weight, crop density, nutrient management, weed management, growth regulators, harvest and postharvest management, and drying conditions [49,72]. Finally, crocin (digentiobiose ester of crocetin) is recognized as a natural food-grade dye that displays biological activity such as antigenotoxic, cytotoxic, antioxidant, anti-inflammatory, anti-atherosclerotic, anti-diabetic, hypotensive, hypolipidemic, hypoglycemic, and antidepressant properties [14,28,55].

Crocetins are lipophilic carotenoids derived from the hydrolysis of crocin glycosides, which is a crocin aglycone [67]. It contains a carboxyl group at each end of the polyene chain [19]; these groups of compounds (α-crocetin or crocetin I, crocetin II, β-crocetin, γ-crocetin) are produced from the degradation of zeaxanthin [73].

Picrocrocin’s structure was established by Khun and Winterstein in 1934 [60]. It is a colorless and odorless glycoside monoterpene (4-hydroxy-2,6,6-trimethyl-1-cyclohexene-1-carboxaldehyde or hydroxy-β-cyclocitral: HTCC and glucose), a product of the degradation of zeaxanthin, and is responsible for saffron’s bitter taste [8,28,34,52,58,74]. Picrocrocin is the second most abundant component in dry matter content [66,73,75]. During the drying process (35–50 °C for 4–7 h), picrocrocin’s temperature and/or hydrolysis form an aglycone [73,76]. Therefore, picrocrocin decreases during dehydration, whereas safranal is absent before drying [17].

Safranal is an aldehyde monoterpene and the volatile component responsible for saffron essential oil. HTCC (hydroxy-β-cyclocitral or 4-hydroxy-2,6,6-trimethyl-1-cyclohexene-1-carboxaldehyde) is regarded by many authors as a safranal precursor. This compound is obtained by chemical or enzymatic hydrolysis (dissociation) or when the vegetal material is dehydrated and transformed into safranal, but this also happens due to the handling and storage processes [8,53,58,63,75,77,78]. The safranal content changes according to the duration and intensity of drying, causing quality fluctuations [34], whereas its concentration increases with the storage and timely harvesting of flowers. However, heat and sunlight decrease the final quality and price [28].

### 5.2. Hypotheses on the Method of Obtaining Apocarotenoids

**Figure 1:**
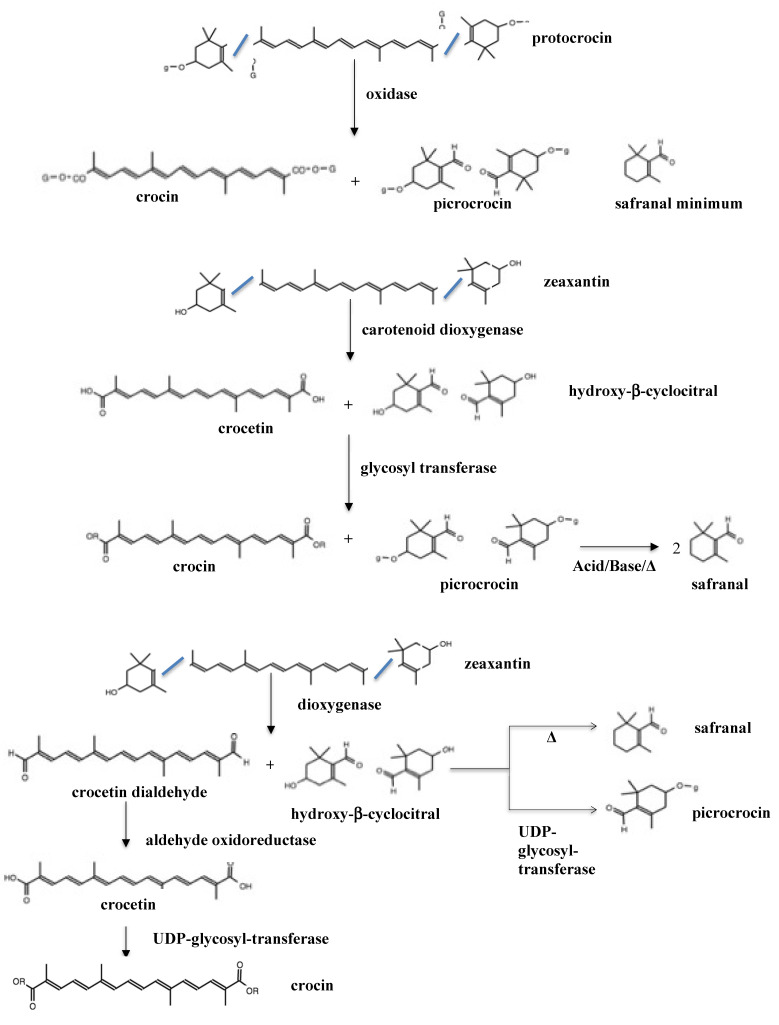
Possible pathways of commercial apocarotenoids in saffron.

There are various hypotheses on the method of obtaining these important apocarotenoids from saffron. The first theory focuses on synthesizing these compounds in the plant from protocrocin (glycosyl derivative of zeaxanthin), the substrate of an oxidative enzyme that produces a molecule of crocin and two molecules of picrocrocin. Regarding safranal, it has been described that only a minimal concentration is detected in the fresh spice [79]. Fallahi et al. [80] described another pathway wherein apocarotenoids, which are commercially important, are obtained by the cleavage of carotenoids (zeaxanthin and β-carotene) by the carotenoid dioxygenase enzyme, giving rise to crocetin and hydroxy-β-cyclocitral as products. Later, they propose a glycosylation (glycosyltransferases) step, which produces crocins and picrocrocin, respectively. Finally, they describe that picrocrocin is hydrolyzed to form safranal. This hypothesis is consistent with that described by Sereshti et al. [81], who also describe other, more specific enzymes and substrates, as seen in Figure 1.

The enzyme dioxygenase performs a 7–8C and 7′–8′C symmetric cleavage on the carotenoid zexanthin, converting it to 3-hydroxy-𝛽-cyclocitral and dialdehyde crocetin. Crocetin dialdehyde undergoes oxidation by aldehyde dehydrogenase to crocetin. Crocetin further undergoes glycosylation at the carboxyl group by the enzyme UDP-glucuronosyl transferase, forming crocin. Picrocrocetin is obtained from 3-hydroxy-𝛽-cyclocitral by glycosylation at the hydroxyl group by the enzyme UDP-glucuronosyl transferases. Picrocrocin is converted to safranal by the action of the enzyme 𝛽-glucosidase along with heat during drying [14].

## 6. Saffron Quality: Compounds Related to Color, Odor, and Flavor

Saffron’s quality depends on its chemical profile, which provides the bitter taste, desirable aroma, and attractive yellowish-red color of this spice [29,82]. Several studies on saffron stability are related to temperature, humidity, pH, light, oxygen [76], geographical growth location, and drying and storage conditions [83]. Since 1980, a standard quality procedure has been employed for saffron classification according to the International Standard Organization (ISO/TS 3632), which was updated in subsequent years (2003, 2010, 2011). This regulation allows saffron to be classified into distinct categories based on physical and chemical criteria: Category I—high quality; Category II—±medium quality; and Category III—low quality [61,84,85]. The grouping parameters used are moisture content, flower residues, foreign material, ash, and coloring power. However, external parameters, such as the absence of other plants, biological micro-flora, and pesticide residues, are also used. The methodology to determine saffron’s quality using these regulations is the spectrophotometric quantification of the stigmas’ aqueous extracts (1%) at three maximum wavelengths, namely 257 nm to indicate flavor strength (picrocrocin), 330 nm related to aroma (safranal), and 440 nm for coloring force (crocins), using a 1 cm pathway quartz cell [85,86,87,88,89]. The results are reported according to Equation (1):

(1) $$E_{1cm}^{1\%}\left(\lambda max\right)=\frac{\left(A\times 10,000\right)}{m\times \left(100-H\right)}$$

where *λmax* is the wavelength (257, 330, or 420 nm), *A* is the absorbance, *m* is the saffron sample weight (g), and *H* is the moisture content (%) [20,79,88,90,91,92]. The color intensity is the most important characteristic related to quality and is used to establish the market price of saffron [93]. The crocin content (degraded carotene) [32] determines the market color specifications. Category I includes a minimum value of 200 units of coloring strength (ucs) and for Category III, the minimum value is 120 ucs [61]. Saffron merchants usually consider a 3-4-year shelf life for saffron when stored under suitable conditions (at room temperature without light exposure). The color intensity decreases by nearly 30 to 40 units per year and is a significant determinant of the final quality of saffron [94]. Diverse drying methods affect crocins, which may be related to the time, temperature, and resistance used [35]. Other factors that affect color are geographic location, harvest, storage, and mixing with additional non-colored parts of the plant (stems and other adulterating materials) [91]. Saffron’s bitter taste is attributed to picrocrocin, a compound present in the plant’s stigmas. The ISO standard determines the flavor strength with values of 70 (Category I), 55 (Category II), and 40 (Category III) [61]. The final picrocrocin content varies according to the dehydration process used [94]. The spice’s flavor can suffer significant losses during processing [1]. Safranal is the active odor in this spice [18,94,95]. The ISO 3632 method determines three categories of aroma strength in safranal, with values within a range of 20–50 [61,96]. It is important to emphasize that during dehydration and storage, there are modifications in saffron’s sensory characteristics [94,97]. 

Therefore, the chemical components of saffron quality are crocin, picrocrocin, and safranal. Lage and Cantrell [21] established that crocins are found in a more significant range (18–37%), followed by picrocrocin (4.2–28%) and, in a lower proportion, safranal (0.04–0.48%). This is consistent with the results described by various authors [21,64,72,90,96], who determined crocins as the major components, specifically trans-4-GG and trans-3-Gg crocins [61,64,98]. 

Concerning crocins, Chaouqi et al. [87] demonstrated that these coloring components are extracted in a more considerable proportion at 40 °C than at room temperature; the authors suggested the use of short dehydration times since an increase in temperature allows for the maximum crocin content, which also depends on the production [94]. However, Rocchi et al. [68] found that the use of elevated temperatures (125–200 °C) in the drying treatment can influence the pigments’ degradation (glucose hydrolysis), and fresh samples (<1 year) retain a significant amount of glycosylated crocin, which is hydrolyzed after storage. Sereshti et al. [81] described that freshly dried samples have an intense color due to crocins since during storage, these pigments decrease (enzymes, temperature, light, hydrolysis), with a negative correlation with odor (the color is reduced, whereas the aroma increases). Saffron storage causes apocarotenoids’ glycosidic bonds to break down (band at 1028 cm), which was confirmed using FT-IR spectroscopy, and is associated with the presence of glucose, together with intensities in the region of 1175–1157 cm linked with glucosidic bonds [99]. The second quality component in the percentage is picrocrocin, which increases with the dehydration temperature (40 °C) [21] but decreases with storage time [87]. Ordoudi et al. [78] determined that saffron produced under optimal processing and storage conditions retains its organoleptic characteristics for 1 to 4 years. Meanwhile, samples stored for more than four years produce low amounts of crocetin and picrocrocin esters. This is related to the findings described by Sereshti et al. [81], who determined that during storage, picrocrocin loses its sugar residues and becomes HTCC and safranal (fresh samples are more bitter). In other words, fresh samples contained a higher concentration of crocins and picrocrocins, whereas the level of safranal (the most abundant volatile component, but with a minimum total concentration in the aromatic spice) was higher in the stored samples; therefore, the relationship between time and safranal content was demonstrated by the higher concentration in the samples with extended storage. García-Rodríguez et al. [96] determined that the aged spice produces safranal from HTCC. The safranal concentration depends on the drying and storage conditions [97].

### 6.1. Quality Standards and Apocarotenoid Quantification

The ISO standard proposes a fast, economical, and easy-to-implement spectrophotometric UV-vis method for aqueous saffron extracts. However, this technique does not allow for the actual determination of the quality compounds [87]. ISO 3632 proposes the quantifications of picrocrocin, safranal, and crocins at a maximum of 257 nm, 330 nm, and 440nm, respectively. However, Cossignani et al. [88] and Aiello et al. [86] determined that crocins show an absorption spectrum between 250 and 470 nm that overlaps at various wavelengths between the compounds. Trans-crocin isomers showed two bands: the first at 260 nm (glycosidic ester bond) and the second band between 400 and 470 nm (typical of carotenoids). Meanwhile, the cis-crocin isomers showed three bands: two bands as previously described and a third band of medium intensity at 328 nm. This indicates that the amount of picrocrocin is affected by the concentration of cis and trans-crocins. Meanwhile, the safranal concentration obtained by UV-vis is not precise since cis-crocins interfere. In summary, overlapping causes quantification errors and limitations in this technique [57,90,96,100,101,102]. Another group of compounds that could interfere with saffron’s quality is the kaempferol derivatives, which absorb UV-vis light at 264 and 344 nm [88,103]. Moreover, safranal is slightly soluble in water and therefore the use of hexane and chloroform has been determined as the best strategy for the extraction and detection of adulterants [101,104].

### 6.2. Apocarotenoids and Their Quantification by Chromatography

Color, flavor, and odor are the quality parameters for saffron aqueous extract according to ISO 3632. They are determined by a non-specific spectrophotometric technique, albeit with limitations in assessing the authenticity of saffron. In the search for a more effective technique, liquid chromatography (LC) or HPLC have been proposed to separate and identify the components contained in a sample [89]. Various studies have described the identification and detection of saffron metabolites by HPLC including safranal, crocins, picrocrocin, and kaempferol and its derivatives [86]. For its part, a mass spectrometry (MS) detector coupled to HPLC and/or DAD could improve quantification [105,106], and MS/MS could facilitate the identification of compounds through structural elucidation [107]. The key quality parameter of saffron is color and the compound to which it is attributed is crocin, which must be quantified in order to determine the market price. For the qualitative and quantitative determinations of crocins, it is necessary to implement standards (quantification by internal and external standards) such as trans-4-GG-crocin (high price and questionable purity ~80%) [53,67,102].

The MS detector has been of considerable help since the lack of suppliers and the high prices of the standards make the structural elucidation (fragmentation patterns) of each crocin important (the different crocins can be identified by the number of hexoses and the molecular weight provided by the mass spectra) to compare them with the patterns in the scientific literature [102,107]. Crocin determination was carried out by Aghhavani et al. [28]; they determined no correlation between the color indexes obtained with spectrophotometry and HPLC data. They concluded that one could use the most accurate, easiest, and low-cost method depending on the experimental conditions to evaluate the quality of saffron. Rocchi et al. [68], demonstrated a poor correlation between the total crocin content (quantification) obtained by the ISO method and by UHPLC-MS/MS.

García-Rodríguez et al. [96] and Kabiri et al. [90] found that the quantification of safranal obtained by UV-vis does not correlate with HPLC data due to the interferences (overestimation by interference) generated by cis-crocetin esters and other compounds with *λmax* 330 nm. They also demonstrated that crocins interfere with picrocrocin and safranal, resulting in overestimates of the latter compounds in samples with large amounts of crocin. They concluded that semipreparative HPLC could represent an efficient method for the quantification of apocarotenoids. Similar results were presented by Moras et al. [106]; they reported that safranal content is more accurately calculated using UHPLC-DAD-MS because it is not influenced by the overestimation of safranal (with cis-crocetin esters at *λmax* 310–330 nm), which is shown when using the ISO methodology. They recommend determining, separating, identifying, and quantifying the metabolite content using the UHPLC-DAD-MS method as a unique and rapid analysis technique. Maggi et al. [104] and Bononi et al. [100] reported a null correlation between safranal content obtained by ISO 3632 and the GC method, as many other saffron substances display absorbance at a maximum of 330 nm.

For this reason, several instruments and analytical methods have been developed for saffron quality control, including chromatography, spectroscopy, molecular biology, and biomimetic techniques, with varying degrees of success and benefits [89]. HPLC is used to isolate, identify, quantify, purify, and determine the quality or adulteration; reverse-phase chromatography is widely used as it is capable of detecting compounds of different polarities and molecular masses [108]. Some authors have pointed out that HPLC-DAD is a selective, precise, sensitive, and specific technique that could evaluate the commercial quality of saffron [27,109]. 

In Table 1, the major commercial-quality compounds in saffron quantified by HPLC, are shown. The extractant solvents used in the investigations (Table 1) are polar and are in agreement with the descriptions by Rahaiee et al. (2015), who suggested that solvents such as water, ethanol, and pure methanol can be used but that mixtures would be more appropriate for the extractions of bioactive compounds [110]. For many authors, ethanol is the most suitable solvent (compared to methanol, ethyl acetate, diethyl ether, hexane, and/or water) for extracting metabolites from saffron stamens [111]. Meanwhile, Rahaiee et al. (2015) showed that an ethanolic extract obtained higher yields compared to water and methanol [66]. Similarly, this solvent was better than methanol for obtaining qualitative and quantitative data from saffron extracts. Meanwhile, Kyriakoudi et al. (2012) recommended the mixture of methanol: water (1:1, *v*/*v*) as a suitable solvent for industrial and analytical applications of saffron apocarotenoids [112]. Crocin isolation by solubility in a water–organic solvent mixture was tested by Zhang et al. (2004), who showed better results for methanol–water > ethanol–water > acetone–water extract [113]. Crocins are the most determined compound, followed by picrocrocin and safranal. In crocins, the ratios determined from highest to lowest were trans-4-GG, trans-3-Gg, cis-4-GG, trans-2-G, and trans-2-gg, respectively. An exception was Moratalla-López et al. [109], whose results did follow this relationship because the saffron samples used in their research were only of quality grade III. In general, ISO 3632 is used by researchers as a preliminary test. However, to perform the true quantification of saffron’s commercial-quality compounds, more precise spectroscopic techniques are used (HPLC, GC-MS, etc.). 

## 7. Saffron Authentication

Due to its high market price, saffron is the most adulterated spice in history, which is most frequently carried out by adding adulterants such as pulverized stigmas [114,115] since diverse plants with similar color and morphology to saffron function as adulterants when mixed [86]. Saffron adulteration can be classified into five common practices, as follows: (1) Adulteration using material from other plants such as calendula, arnica, gardenia, beet, pomegranate, turmeric, achiote, and safflower [93,106,115,116] or with other plant parts of *C. sativus* besides the stigmas; (2) Increasing saffron mass by moistening with honey, corn silk, sugar, fat, inorganic compounds, vegetable oils, or glycerin [18,116]; (3) Using natural or artificial food-grade colorants such as tartrazine, ponceau-4R, quinoline, methyl orange, sunset yellow, Sudan II, and Allura red [117,118]; and other less-used adulteration methods including (4) The addition of exogenous components mixed with food flavorings (erythrosine) and extracted spent saffron (recolored or old), and (5) Geographic origin tagging fraud [31,93,119,120]. 

The chemical composition of food is an indicator of quality, origin, authenticity, and/or adulteration. The chemical profile, also known as spectral fingerprinting or chemo typing, is considered a characteristic pattern [121]. In food, variations in a profile are related to alterations in production systems, the geographical origins of raw materials, storage conditions, or adulterant practices [122]. It should be emphasized that it is important to identify the adulterant and quantify the adulteration level [123]. Furthermore, the ISO/TS 3662 spectrophotometric technique does not differentiate between genuine and adulterated saffron [9,124]. Saffron authentication is based on a pharmacognostic analysis (microscopic examination of histomorphological features). It is time-consuming and requires the availability of trained and experienced personnel [115,125]. 

Regulatory systems evaluate saffron using sensory inspections (macroscopic and microscopic examinations) as well as conduct quantitative determinations of specific chemical compounds [126]. Authentication is based on detecting known chemical compounds obtained with instrumental signals [127]. However, these kits yield many characteristics or compounds, making it necessary to establish the chemical markers of authenticity [128]. Spectral fingerprinting can also detect and quantify adulterations using statistical data [127]. Chemometrics uses mathematical and statistical methods to create a correlation between the sample properties and chemical data obtained from analytical instruments [129]; this area is based on optimizing the experimental design and extracting useful information from large and complex data sets [122]. Therefore, analytical chemometric coupling could notably decrease the number of characteristics/compounds/signals and generate the markers responsible for different authenticity issues (adulteration detection, variety or geographical origin, discrimination, organoleptic profile, maturation, and production method). In addition, the identified markers would help to establish databases containing complete and standardized information on the chemical profiles [128]. 

The following research summary is based on determining chemical compounds as authentication markers (of genuine saffron or adulterants used) using different analytical techniques to determine the spectral fingerprints and/or even using chemometrics to obtain the amount of the adulterant or even the detection limits of the adulterant. Saffron adulteration determination by the inclusion of tepals and/or stamens was carried out by Senizza et al. [9]. They determined 232 compounds using UHPLC-QTO-MS. Among them, 77 chemicals were present in trace quantities including the presence of flavonoids: 11 flavanols (tepals had a high content) and 7 anthocyanins (pigments of flowers, fruits, and other plant organs), which increased in the adulterated samples. On the other hand, lignans (12 compounds) were found in low amounts in the authentic samples. Zeaxanthin and picrocrocin, which decreased in the adulterated samples, suggested a possible “dilution effect” when adding adulterants. Moras et al. [106] determined, through UHPLC-DAD-MS, the presence of iridoids as a marker for saffron adulteration, yielding positive test results when gardenia extract was added. 

Investigations using analytical techniques and chemometrics to quantify the adulterant and the minimum detection to detect fraud have been presented. A method for deducing saffron authenticity using LC-MS with derivatives of kaempferol and geniposide was developed by Guijarro-Díez et al. [119]. They detected a minimum quantifiable value of adulteration (0.2%) regardless of the adulterant (linear regression lineal and ANOVA), the specific method, and saffron quality control. Sabatino et al. [85] used HPLC-PDA-ESI-MS to identify unusual concentrations of adulterants in saffron (10–67% safflower, calendula, and turmeric). Their results showed that the ISO did not detect the addition of 10% of adulterants. Moreover, marker molecules such as picrocrocin, trans-5-nG, trans-4-GG, trans-4-ng, cis-3-Gg, cis-4-GG, and cis-2-gg were not found in the adulterated spices. They determined the addition of 5% of safflower or calendula and 2% addition of turmeric in the analyzed samples. 

Saffron stigma adulteration with up to 20% of plant derivatives (saffron stamens, calendula, safflower, turmeric, buddleja, and gardenia) was determined by Petrakis and Polissiou [123] using a DRIFTS method and chemometric techniques. PLS-DA was applied to perform saffron authentication based on infrared fingerprints (4000–600 cm). Identification was carried out with data from the 2000–600 cm^−1^ region to develop the mathematical models and detection limits ranging from 1.0 to 3.1% (p/p). Another (NIR) spectroscopy investigation combined with multivariate data analysis was performed by Shawky et al. [130]. They performed saffron stigma authentication with other plants (safflower, pomegranate peel, calendula flower, paprika, turmeric, hibiscus, saffron stamens, and re-extracted saffron stigma), modeling them with data at the spectral region (9000–4000 cm^−1^). The use of PLS-DA allowed them to differentiate between authentic, adulterated, and mixed adulterant samples, with a detection limit of up to 10 mg/g of the adulterant. In addition, they quantified other added adulterants. 

Saffron stigma authentication using artificial intelligence (simulating senses: sight, smell) was reported by Heidarbeigi et al. [7]. They determined plant adulterants (safflower and dyed corn using beetroot as a colorant, in addition to their mixtures) through signals obtained by the e-nose (managing to differentiate adulterated and unadulterated saffron). They also applied PCA and artificial neural networks (ANN) to determine fraud in saffron stigmas, determining adulteration levels higher than 10%. Kiani et al. [83] used CVS (camera, lighting system, and software) and an e-nose in combination with multivariate methods (PCA, HCA, and SVMs) to detect saffron stigma adulterants (colored safflower and saffron style) based on color and aroma profiles. The test demonstrated the ability to identify the adulterated samples and this was achieved using ANN-MLP models, concluding that neural networks allowed color (89%) and aroma-intensity (100%) prediction. CVS was used by Minaei et al. [91] to characterize saffron color by sample image analysis. The use of PCA to group color characteristics and the use of PLS, MLR, and MLP neural networks (color characteristics used: R, Y, I, and Cr) related color and dye force (ISO 3632), with a correlation coefficient of 0.89 and a success rate of 96.67%. 

Another interesting application is the use of an e-nose (non-conventional technique), compared to IR-MS and GC-MS (conventional techniques) to discriminate among saffron samples with different origins, ages, and types of drying. The e-nose, in conjunction with PLS-DA, was able to discriminate between samples of saffron with different origins; this unconventional methodology was proposed to detect adulterates [131]. Recently, molecular techniques for detecting fraud by adulterations have gained interest. Safflower adulteration stamens as saffron adulterants were also studied by Babaei et al. [124], using a multiplex PCR technique. Khilare et al. [116] described three methods to achieve saffron authentication (microscopic examination, ISO3632 standard, and DNA barcode). They evaluated 36 saffron samples and showed that the ISO only determines the color and aroma, while the microscopic method indicates color purity and uniformity (possible adulterants).

Finally, DNA codes (gene code used: rbcL) have allowed researchers to authenticate saffron’s origin and quality. Torelli et al. [115] used SCAR to detect adulteration or contamination. SCAR markers can represent a rapid, reliable, and inexpensive method for saffron authentication. Other rapid techniques for determining saffron adulteration were proposed by Zhao et al. [132] via DNA extraction. They used a recombinase polymerase amplification (RPA-LFD), which allowed them to perform the rapid visual detection of the saffron and adulterated samples. Finally, when saffron was immersed in water, it expanded immediately; when a diphenylamine and sulfuric acid solution was added, the saffron was colored with a blue tone and quickly became reddish brown. Saffron phenylethanol varies according to the spice preparation and is related to the stamen pollen [93]. Table 2 shows a summary of the various research works and techniques for the determination of the different types of adulterants. As regards the adulteration of saffron by its origin or PDO products, saffron has a high value on the market so some saffron producers falsify the product’s origin [15,54]. In Europe, a PDO label carries a regional valuation that identifies the products produced, processed, and prepared in a specific geographic area [103]. There are five brands recognized with this label: “Krokos Kozanis” (Greece), “Azafrán de la Mancha” (Spain), “Zafferano dell ‘Aquila”, “Zafferano di San Gimignano”, and “Zafferano di Sardegna” (Italy) [15]. There have been a considerable number of studies on origin adulteration [31,54,101,103,131,133,134,135]. La Mancha in Spain and Kashmir in India are two regions where saffron maintains higher prices [134]. Therefore, labeling saffron samples with a PDO implies that the product is of high quality [54]. Moreover, Senizza et al. [9] determined the chemical markers capable of discriminating PDO saffron samples from non-PDO. Chemical fingerprints were obtained using UHPLC-ESI-QTOF-MS and multivariate statistics, obtaining the flavonoids belonging to the flavonols and flavones (pelargonidin 3-O-6-succinyl-glucoside, isoxanthohumol, nobiletin, jaceosidin, 6-hydroxyluteolin, 3-methoxysinenset, 7-dimethylquercetin, quercetin 6-O-malonylglycitin), phenolic acids (protocatechuic aldehyde, 4-hydroxybenzaldehyde, vanillin, 2/3/4-hydroxybenzoic acids, benzoic acid, sinapine, p-coumaroyl malic acid, p-coumaric acid, cinnamoyl glucose, 4-hydroxyphenylacetic acid), lignans, and other polyphenols. 

A suitable method is the use of NMR in conjunction with multivariate statistical analysis. Principal component analysis allowed the discrimination between the samples of Italian PDO and commercial saffron, despite the year of harvest, date of purchase, and storage time [101]. Bosmali et al. [15] proposed a molecular approach for the authentication of the “Krokos Kozanis” brand using specific ISSR (inter-simple sequence repeat) markers to evaluate the variability within the *C. sativus* L. species (differences in bands produced by other *Crocus* species). The species-specific markers such as HRM analysis were developed in conjunction with the DNA barcode regions.

## 8. Saffron By-Products

The preparation of saffron is expensive due to the intense harvesting work and postharvest processes (dehydration and storage) required [32]. It is known that in order to produce 1 kg of stigma, around 1000 kg of flowers are treated by weight, which represents 220,000–260,000 flowers [42,98]. Therefore, saffron cultivation is not highly profitable in terms of biomass, which increases the interest in minimizing losses and ensuring efficient waste management [140]. Several reports have focused on the stigma, which is the plant’s biologically active part [141]; its bioactivity is attributed to the composition, containing the main chemical components and their synergy with other compounds [60]. 

However, the by-products are also important since their use could increase the *C. sativus* flower’s economic value, considering that other parts of the plant contain compounds with sensorial properties or biological activity [98,140]. *C. sativus* tepals are the main by-product of saffron production [142] but the flowers have low safranal content so they cannot be consumed or sold as saffron on the spice market [42]; only the leaves are used as forage [143]. Using HPLC-DAD, Serrano-Díaz et al. [144] determined kaempferol 3-Osophoroside and delphinidin 3,5-di-O-glucoside as the main components of the aqueous by-products of saffron flowers. Tepal and stamen biomarkers were determined by Mottaghipisheh et al. [145] using HPLC-DAD; they reported crocin, crocetin, picrocrocin, safranal, kaempferol-3-O-sophoroside, kaempferol-3-O-glucoside, and quercetin-3-O-soforoside. Tepal’s main component was kaempferol-3-O-sophoroside with crocin, crocetin, and picrocrocin; safranal was not detected in any of the analyzed samples. Table 3 shows the principal agro-industrial by-products of saffron that have been investigated and their possible uses. Lahmass et al. (2017) determined that the corms, leaves, and spasms of *C. sativus* may possess anti-aging or anticancer properties.

These investigations generate interest in valorizing the various parts of saffron flowers and improving small-scale farmers’ incomes. These results could contribute to the development of innovative products from saffron flowers and more effective biological waste management and exploitation [146]. It is important to emphasize knowledge of the components’ depth (majority or minority) within each potentially valuable plant part of the saffron plant, which could help in determining the most suitable application [10].

## 9. Conclusions

The high commercial value of saffron is a result of the production (harvesting, drying, and storage) and low biomass yield, a critical characteristic of market fraud. Saffron is used in the food industry as an aromatic species to give flavor, color, and odor to various foods, but its extracts or extractive compounds are also used as functional ingredients in a large number of products (desserts, beverages, oils, pastes, etc.). The ISO 3632 standard proposes a spectrophotometric technique for the determination of the commercial quality of saffron. This methodology has the great advantage of being easy to prepare, accessible, and low-cost in terms of equipment. The quality of *C. sativus* is based on the quantity of the main apocarotenoids (crocin, picrocrocin, and safranal). However, for the quantification of saffron apocarotenoids, more rigorous, sensitive, selective, and related analytical techniques (UHPLC/QTO/MS, DRIFTS, NIR, SCAR, PCR, etc.), which provide more accurate concentrations, are preferred. Moreover, the results obtained by spectrophotometry yield inaccurate results (overlapping of chemical compounds, poor solubility of safranal, erroneous quantification of compounds, and non-identification of adulterants). Therefore, the ISO standard is only proposed as a preliminary methodology to rule out low-quality saffron and is not suitable for authentication and/or the detection of adulterants. The determination of the chemical profiles or fingerprints of the sample or aromatic plant is used to obtain the markers of the saffron or adulterants. These signals or fingerprints obtained by analytical techniques coupled to chemometric methods (principal component analysis, linear discriminant analysis (LDA, etc.) favor the discrimination of adulterated samples, possible adulterant plants or compounds, the detection limits of the equipment, and even the concentrations at which they are obtained. Finally, it was determined that not only the stigmas contained bioactive compounds since this work describes some research on saffron flower by-products that contain a large number of phytochemical compounds (catechin, quercetin, delphinidin, etc.). For these reasons, saffron is an interesting and aromatic spice as a colorant, antioxidant, and source of phytochemicals.

## Figures and Tables

**Table 1:** Principal quality chemical components of saffrons obtained from different geographical origins and their concentrations.

Geographical Origin	Type of Extract	Compound	Concentration		Technique	Ref.
Azerbaijan	Methanol–water (50:50, *v*/*v*)	Trans-4-GG	39.08	mg/g	HPLC-PDA	[53]
		Trans-3-Gg	27.25			
		Cis-4-GG	7.49			
		Σ crocins	77.16			
		Picrocrocin	3.34			
		Safranal	0.98			
China	Methanol–water (50:50, *v*/*v*)	Trans-4-GG	6.29	mg/g	HPLC-PDA	[53]
		Trans-3Gg	2.44			
		Σ crocins	8.73			
		Picrocrocin	0.53			
		Safranal	0.22			
Poitou, France	Methanol–water (50:50, *v*/*v*)	Trans-4-GG	38.43	mg/g	HPLC-PDA	[53]
		Trans-3-Gg	27.74			
		Cis-4-GG	5.89			
		Σ crocins	75.07			
		Picrocrocin	5.97			
		Safranal	0.81			
Greece	Methanol–water (50:50, *v*/*v*)	Trans-4-GG	40.77	mg/g	HPLC-PDA	[53]
		Trans-3-Gg	30.36			
		Cis-4-GG	10.14			
		Σ crocins	86.51			
		Picrocrocin	5.95			
		Safranal	1.29			
India	Methanol–water (50:50, *v*/*v*)	Trans-4-GG	37.54	mg/g	HPLC-PDA	[53]
		Trans-3-Gg	22.13			
		Cis-4-GG	9.12			
		Σ crocins	75.68			
		Picrocrocin	7.87			
		Safranal	0.47			
Fars, Iran	Aqueous extracts	Trans-4-GG	56.16	mg/g	HPLC-DAD	[27]
		Trans-3-Gg	48.72			
		Cis-4-GG	12.53			
		Trans-2-gg	12.49			
		Σ crocins	153.81			
		Picrocrocin	77.29			
Ghaen, Iran	Ethanol (70%)	Trans-4-GG	197.84	mg/g	HPLC-DAD-MS	[64]
		Trans-3-Gg	71.56			
		Cis-4-GG	26.88			
		Trans-2-G	24.86			
		Σ crocins	338.87			
		Picrocrocin	43.82			
		Safranal	1.35			
Gonabad, Iran	Ethanol (70%)	Trans-4-GG	168.91	mg/g	HPLC-DAD-MS	[64]
		Trans-3-Gg	61.25			
		Cis-4-GG	30.42			
		Trans-2-G	26			
		Σ crocins	302.51			
		Picrocrocin	36.97			
		Safranal	1.26			
Isfahan, Iran	Aqueous extracts	Picrocrocin	150.64	mg/g	HPLC-DAD	[27]
		Trans-4-GG	46.86			
		Trans-3-Gg	43.51			
		Trans-2-G	14.53			
		Trans-2-gg	10.56			
		Σ crocins	137.05			
		Safranal	1.04			
Kerman, Iran	Aqueous extracts	Trans-4-GG	77.89	mg/g	HPLC-DAD	[27]
		Trans-3-Gg	46.69			
		Trans-2-G	12.79			
		Σ crocins	159.86			
		Picrocrocin	63.95			
		Safranal	1.31			
Razavi Khorasan, Iran	Aqueous extracts	Trans-4-GG	54.73	mg/g	HPLC-DAD	[27]
		Trans-3-Gg	34.51			
		Trans-2-G	9.35			
		Σ crocins	123.61			
		Picrocrocin	120.62			
		Safranal	2.13			
Tehran, Iran	Aqueous extracts	Trans-4-GG	59.7	mg/g	HPLC-DAD	[27]
		Trans-3-Gg	44.43			
		Cis-4-GG	12.39			
		Trans-2-gg	9.34			
		Σ crocins	146.66			
		Picrocrocin	131.61			
		Safranal	0.57			
Tehran, Iran	Aqueous extracts (1%) Freeze-Dried	Picrocrocin	33.88	mmol/100g	HPLC-DAD	[109]
		HTCC	20.2			
		Trans-3-Gg	3.81			
		Trans-4-GG	3.53			
		Trans-2-gg	1.17			
		Σ crocins	9.91			
		Safranal	0.84			
Tehran, Iran	Aqueous extracts (1%) Dark-Dried	HTCC	16.82	mmol/100g	HPLC-DAD	[109]
		Picrocrocin	15.14			
		Trans-4-GG	4.59			
		Trans-3-Gg	3.71			
		Σ crocins	11.95			
		Safranal	0.41			
Torbat, Iran	Ethanol (70%)	Trans-4-GG	238.02	mg/g	HPLC-DAD-MS	[64]
		Trans-3-Gg	85.36			
		Trans-2-G	24.3			
		Cis-4-GG	19.38			
		Σ crocins	388.23			
		Picrocrocin	67.95			
		Safranal	1.79			
Iran	Aqueous extracts	Trans-4-GG	42.24	%	HPLC	[70]
		Trans-3-Gg	24.76			
		Cis-4-GG	5.09			
		Trans-2-G	3.53			
		Trans-2-gg	3.18			
		Σ crocins	83.06			
		Picrocrocin	16.72			
		Safranal	0.22			
Iran	Methanol–water (50:50, *v*/*v*)	Trans-4-GG	38.41	mg/g	HPLC-PDA	[53]
		Trans-3-Gg	23.58			
		Cis-4-GG	4.73			
		Σ crocins	69.32			
		Picrocrocin	3.69			
		Safranal	0.65			
Iran	Ethanol 80%	Crocin	26.81	mg/0.1g	HPLC	[90]
		Picrocrocin	12.92			
		Safranal	0.042			
Cascia, Italy	Ethanol (70%)	Trans-4-GG	343.97	mg/g	HPLC-DAD-MS	[64]
		Trans-3-Gg	111.94			
		Trans-2-G	13.59			
		Σ crocins	494.42			
		Picrocrocin	127.83			
		Safranal	3.01			
Città della Pieve, Italy	Ethanol (70%)	Trans-4-GG	302.65	mg/g	HPLC-DAD-MS	[64]
		Trans-3-Gg	109.17			
		Trans-2-G	16.12			
		Σ crocins	450.73			
		Picrocrocin	101.92			
		Safranal	2.41			
Fiesole, Italy	Ethanol (70%)	Trans-4-GG	372.49	mg/g	HPLC-DAD-MS	[64]
		Trans-3-Gg	123.15			
		Trans-2-G	21.24			
		Cis-4-GG	12.55			
		Σ crocins	548.84			
		Picrocrocin	130.35			
		Safranal	2.01			
Fiesole, Italy	Ethanol (70%)—formic acid	Trans-4-GG	238.91	mg/g	HPLC-DAD-MS	[98]
		Trans-3-Gg	65.64			
		Trans-2-G	16.96			
		Cis-4-GG	4.95			
		Σ crocins	342.02			
		Picrocrocin	111.14			
		Safranal	2.27			
Navelli, Italy	Methanol–water (50:50, *v*/*v*)	Trans-4-GG	38.25	mg/g	HPLC-PDA	[53]
		Trans-3-Gg	28.28			
		Σ crocins	72.02			
		Picrocrocin	5.8			
		Safranal	0.53			
Perugia, Italy	Ethanol 70%—formic acid	Trans-4-GG	148.5	mg/g	HPLC-DAD-MS	[98]
		Trans-3-Gg	46.2			
		Trans-2-G	14.8			
		Cis-4-GG	14.1			
		Σ crocins	231.1			
		Picrocrocin	68.9			
		Safranal	2.6			
Italy	Aqueous extracts	Trans-4-GG	43.57	%	HPLC	[70]
		Trans-3-Gg	23.09			
		Cis-4-GG	5.29			
		Trans-2-gg	2.12			
		Σ crocins	78.45			
		Picrocrocin	21.26			
		Safranal	0.28			
Larache, Marruecos	Degassed methanol	Σ crocins	17.9	%	HPLC-DAD	[21]
		Picrocrocin	11.92			
		Safranal	0.21			
Safranier d’Ourika, Marruecos	Degassed methanol	Σ crocins	37.23	%	HPLC-DAD	[21]
		Picrocrocin	28.78			
		Safranal	0.24			
Rangiora, New Zealand	Methanol–water (50:50, *v*/*v*)	Trans-4-GG	41.21	mg/g	HPLC-PDA	[53]
		Trans-3-Gg	31.26			
		Σ crocins	74.61			
		Picrocrocin	7.94			
		Safranal	0.47			
La Mancha, Spain	Methanol–water (50:50, *v*/*v*)	Trans-4-GG	38.41	mg/g	HPLC-PDA	[53]
		Trans-3-Gg	24.43			
		Cis-4-GG	5.76			
		Σ crocins	73.85			
		Picrocrocin	8.14			
		Safranal	0.88			
Turkey	Methanol–water (50:50, *v*/*v*)	Trans-4-GG	36.35	mg/g	HPLC-PDA	[53]
		Trans-3-Gg	25.32			
		Cis-4-GG	5.21			
		Σ crocins	69.73			
		Picrocrocin	5.67			
		Safranal	0.84			

**Table 2:** Different techniques for saffron adulterant determination.

Type	Adulterant	Adulterant Concentration	Adulterant Minimal Detection		Adulterants Indicators or Markers		Technique	Ref.
1. A	Calendula flower Curcuma rhizome Hibiscus flower Paprika fruit Pomegranate fruit Safflower	10–400 mg/g	10	mg/g	6000–5800 5400–5000 4600–4200	cm^−1^	FT-NIR/PCA: SIMCA PLS-DA	[130]
1. A	Gardenia	0–100% *w*/*w*	5	% *w*/*w*	Geniposide Deacetyl-asperuloside acid methyl ester Gardenoside Genipin-1-β-D-gentibioside 6”-O-trans-coumaroylgenipin gentibioside Scandoside methyl ester Absence of picrocrocin derivatives		UHPLC-DAD-MS	[106]
1. A	Gardenia extract	ND	41.7	g/g	Geniposide		LC–MS	[119]
1. A	Gardenia extract	0–100%	0.8 0.2 1.8 2.5 2.2	%	Kaempferol 3,7,40-O-triglucoside Kaempferol 3-O-sophoroside 7-O-glucoside Kaempferol 3,7-O-diglucoside Kaempferol 3-O-sophoroside Kaempferol 3-O-glucoside		LC-MS	[51]
1. A	Curcuma rhizome	0.5–20% *w*/*w*	0.5	% *w*/*w*	ND		DNA isolation/	[15]
							Bar-HRM	
1. A	Calendula Rubia Safflower	5–35% *w*/*w*	5	% *w*/*w*	4200 4750 5170 6000–5400 7100–6000 8300 cm	cm^−1^	NIR/PLS-DA	[117]
1. A	Turmeric, Onion peels Pomegranate peels Calendula petals	0–30% *w*/*w*	3.7 6.2 3.6 3.5	% *w*/*w*	4961–4016 6388–5389 9975–7472	cm^−1^	FT-NIR/MCR-ALS	[136]
1. A	Tumeric Safflower G. jasminoides fruit extract	20% *w*/*w*	20	% *w*/*w*	7.541, 6.751, 6.059, 7.318, 7.147, 6.819 5.205, 5.138, 5.066 7.569, 7.466, 5.679, 5.121	^1^ H ppm	^1^ NMR/OPLS-DA/O2PLS-DA	[125]
1. A	Buddleja Officinalis flower Calendula petals Gardenia fruit extract Safflower Turmeric	0–20% *w*/*w*	1.1–1.6 1.9–2.6 1.1–1.5 2.1–2.8 1–1.6	% *w*/*w*	1624–1456 and 941–771 1508–1396 and 1167–1055 1794–1626 and 1113–943 1539–1456 and 858–773 1624–1286 and 941–771	cm^−1^	DRIFTS/PCA PLS-DA	[123]
1. B	Saffron style	5–35% *w*/*w*	5	% *w*/*w*	4200 4750 5170 6000–5400 7100–6000 8300	cm^−1^	NIR and MIR/PLS-DA	[117]
1. B	Saffron stamens	20% *w*/*w*	20	% *w*/*w*	5.181	^1^ H ppm	^1^ NMR/OPLSDA/O2PLS-DA	[125]
1. B	Saffron stamens	0–20% *w*/*w*	2.2–3.1	% *w*/*w*	4000–600 1963–1626 and 941–771	cm^−1^	DRIFTS/PCA PLS-DA	[123]
1. B	Saffron stamens	10–400 mg/g	10	mg/g	6000–5800 5400–5000 4600–4200	cm^−1^	FT-NIR/PLS-DA	[130]
3	Carminic acid	0.5–20% *w*/*w*	10	% *w*/*w*	1564–1576 1445–1456 1211–1231 810–816	cm^−1^	FT-IR/PCA/PLS-DA	[137]
3	Carminic acid	0.2–2% *w*/*w*	0.2	% *w*/*w*	Carminic acid at 4.7 min, 495 nm	min, nm	RT-HPLC-DAD	[137]
3	Synthetic dyes	ND	Magenta III Rhodamine B		330.1964 (HRMS) 300.14 (EI-MS) 223.11 (EI-MS) 2.5 (HPLC) 443.2320 (HRMS) 399.17 (EI-MS) 316.21 (EI-MS) 3.4 (HPLC)	*m*/*z* min	TLC/EI-MS/HRMS HPLC	[138]
3	Sudan III Sudan I Sudan II Sudan IV	0.14–7.1 g/Kg	0.14	g/Kg	8.014 6.87 8.618 8.181	^1^ H ppm	^1^ H NMR	[139]
4	Exhausted saffron	10–400 mg/g	10	mg/g	6000–5800	cm^−1^	FT-NIR/SIMCA	[130]
					5400–5000		PLS-DA	
					4600–4200			

**Table 3:** Saffron by-products of different geographical origins, major components, and applications.

By-Product (Origin and Type of Extract)	Major Components	Concentration		Application	Ref.
Sepals (Fiesole, Italy; ethanolic)	Trans-4-GG Trans-3-Gg Cis-2-G Kaempferol-3-sophoroside Quercetin diglucoside Kaempferol glucoside Kaempferol sinapoyl glucoside	3.1 0.8 0.2 6.4 0.4 0.4 0.3	mg/g	Phytochemicals	[98]
Stamens (Fiesole, Italy; ethanolic)	Trans-4-GG Trans-3-Gg Cis-4-GG Trans-2-G Kaempferol-3-sophoroside Quercetin diglucoside Methyl quercetin derivative Methyl quercetin diglucoside Kaempferol-3-sophoroside-7-glucoside	112.2 33.4 22.0 20.7 1.7 1.0 0.7 0.6 0.5	mg/g	Phytochemicals	[98]
Sepals (Perugia, Italy; ethanolic)	Traces of crocin Kaempferol-3-sophoroside Quercetin diglucoside Kaempferol glucoside Kaempferol sinapoyl glucoside	nd 8.3 0.7 0.4 0.3	mg/g	Phytochemicals	[98]
Stamens. (Perugia, Italy; ethanolic)	Trans-4-GG	4	mg/g	Phytochemicals	[98]
	Trans-2-G	1.3			
	Methyl quercetin diglucoside	2.1			
	Quercetin diglucoside	1.2			
	Methyl quercetin derivative	1.2			
	Kaempferol-3-sophoroside-7-glucoside	0.9			
	Kaempferol diglucoside	0.8			
Petals (Srinagar, Jammu & Kashmir, India; aqueous)	Not detected			Kashmir dye green and yellow tones	[66]
Petals (Kerman, Iran; aqueous)	Methanol	355	ppb	Volatile compounds in the pharmaceutical industry	[42]
	Biogenix aldehyde fragment	303			
	Acetic acid	492			
	Isobutanal	694			
	Furanone	6397			
	2,3-butanedione	524			
Petals (Sardinia, Italy; aqueous)	Kaempferol-3-O-sophoroside	2790	mg/L	Antioxidant and colon anticancer activities.	[147]
	Phenylalanine	1072			
	Delphinidin 3,5-di-O-glucose	822			
	Tyrosine	619			
	Kaempferol-3,7-di-O-glucoside	368			
	Isorhamnetin-3-O-rutinoside	268			
	Quercetin 3-O-sophoroside	207			
Petals (Northeast, Iran; ethanolic and aqueous)	Pelargonidin 3,5-glycosides	56.1	%	Antioxidant and colorant activities.	[148]
	3,5 cyanidin-diglycosides	20.9			
	Petunidin	15.5			
	Delphinidin 3-glycosides	4.1			
	Pelargonidin 3-glycosides	3.4			
Petals and anthers (Navelli, Italy; ethanolic, oil, and aqueous)	Crocin	0.6	%	Antioxidant and anti-inflammatory (in vivo; in vitro).	[149]
	Catechin	0.2			
	Rutin	0.1			
	Epicatechin	0.08			
	p-OH benzoic acid	0.04			
	Safranal	0.02			
	Vanillic acid	0.02			
	Galic acid	0.09			
	Safranal	0.05			
	Quercetin	0.01			
Petals (Torbat Heydariyeh region, Iran; ohmic extraction)	Crocin	81.2	%	Source of natural flavoring, coloring, and antioxidants.	[41]
	Safranal	5.5			
	Catechin	1.4			
	Epicatechin	1.2			
	Delphinidin 3,5-di-O-glucose	74.2			
	Petunidin 3-O-glucoside	10.3			
	Petunidin 2,5-di-O-glucoside	8.6			
	Quercetin 3-O-glucoside	59.5			
	Kaempferol-3-O-sophoroside	8.2			
	Kaempferol-3-O-glucoside	6.1			
	Quercetin 3-O-sophoroside	5.5			
	Kaempferol	5.4			
Petals. (Torbat Heydariyeh region, Iran; ultrasound extraction)	Crocin	79.02	%	Source of natural flavoring, coloring, and antioxidants.	[41]
	Safranal	4.03			
	Delphinidin 3,5-di-O-glucose	67.88			
	Petunidin 3-O-glucoside	10.74			
	Petunidin 3,5-di-O-glucoside	7.39			
	Quercetin 3-O-glucoside	54.32			
	Kaempferol-3-O-sophoroside	8.16			
	Kaempferol-3-O-glucoside	5.27			
	Quercetin 3-O-sophoroside	5.12			
Petals. (Torbat Heydariyeh region, Iran; microwave extraction)	Crocin	77.42	%	Source of natural flavoring, coloring, and antioxidants.	[41]
	Safranal	5.03			
	Epicatechin	1.02			
	Vanillic acid	1.03			
	Delphinidin 3,5-di-O-glucose	56.36			
	Petunidin 3-O-glucoside	11.44			
	Malvidin O-glucoside	7.94			
	Quercetin 3-O-glucoside	59.49			
	Kaempferol-3-O-sophoroside	8.16			
	Kaempferol-3-O-glucoside	6.13			
	Quercetin 3-O-sophoroside	5.51			
	Kaempferol	5.42			

## Abbreviations

ANN: Artificial Neural Network; ANN–MLP: Multi-Layer Perceptron–Artificial Neural Network; ANOVA: Analysis of Variance; Bar–HRM: Barcode-DNA–High-Resolution Melting; CVS: Computer Vision System; DNA: Deoxyribo Nucleic Acid; DRIFTS: Diffuse Reflectance Infrared Fourier Transform Spectroscopy; EI–MS: Electrospray Ionization–Mass Spectrometry; E-nose: Electronic nose; FT–IR: Fourier Transform–Infrared Spectroscopy; FT–NIR: Fourier Transform–Near-Infrared; GC–MS: Gas Chromatography–Mass Spectrometry; HCA: Hierarchical Cluster Analysis; HPLC: High-Performance Liquid Chromatography; HPLC–DAD: High-Performance Liquid Chromatography coupled with Diode Array Detection; HPLC–DAD–MS: High-Performance Liquid Chromatography coupled with Diode Array Detection–Mass Spectrometry; HPLC–PDA–ESI–MS: High-Performance Liquid Chromatography coupled with Photo Diode Array–Electrospray Ionization–Mass Spectrometry; HTCC: 4-hydroxy-2,6,6-trimethyl-1-cyclohexene-1-carboxaldehyde or hydroxy-β-cyclocitral; HRM: High-Resolution Melting; HRMS: High-Resolution Mass Spectrometry; ISSR: Inter-Simple Sequence Repeat; ISO: International Organization for Standardization; IR–MS: Isotope Ratio–Mass Spectrometry; LC: Liquid Chromatography; LC–MS: Liquid Chromatography–Mass Spectrometry; MCR–ALS: Multivariate Curve Resolution–Alternating Least Squares; MIR: Mid Infrared; MLP: Multi-Layer Perceptron; MLR: Multiple Linear Regression; NIR: Near Infrared; NMR: Nuclear Magnetic Resonance; OPLS–DA: Orthogonal Projection to Latent Structures–Discriminant Analysis; O2PLS–DA: Orthogonal Projection to Latent Structures–Discriminant Analysis with bidirectional modifications; PCA: Principal Component Analysis; PDO: Protected Designations of Origin; PLS: Partial Least Squares; PLS–DA: Partial Least Squares–Discriminant Analysis; PCR: Polymerase Chain Reaction; RPA–LFD: Recombinase Polymerase Amplification in combination with-Lateral Flow dipstick; RT–HPLC–DAD: Reverse Phase–High-Performance Liquid Chromatography coupled with Diode Array Detection; SCAR: Sequence-Characterized Amplified Regions; SIMCA: Soft Independent Modeling of Class Analogies; SVMs: Support Vector Machines; Trans-1-g: crocin-1or trans-crocetin mono-(β-D-glucosyl) ester; Trans-2-gg: crocin-2II, crocin-2′ or trans-crocetin di-(β-D-glucosyl) ester; Trans-2-G: crocin-2 or trans-crocetin (β-D-gentiobiosyl) ester; Trans-3-Gg: crocin-3 or trans-crocetin (β-D-glucosyl)-(β-D-gentiobiosyl) ester; Trans-4-GG: crocin-4 or trans-crocetin di-(β-D-gentiobiosyl) ester); Trans-5-tG: crocin-5 or trans-crocetin (β-D-triglucosyl)-(β-D-gentiobiosyl) ester; TLC: Thin Layer Chromatography; UDP-glucuronosyl transferase: Uridine-diphosphate-glucuronosyl transferase; UHPLC–QTO–MS: Ultra-High-Performance Liquid Chromatography coupled to Quadrupole Time-of-Flight–Mass Spectrometry; UHPLC–ESI–QTOF–MS: Ultra-High-Performance Liquid Chromatography with Electrospray Ionization coupled to Quadrupole Time-of-Flight–Mass Spectrometry; UHPLC–DAD–MS: Ultra-High-Performance Liquid Chromatography with Diode Array Detection–Mass Spectrometry; UHPLC–MS/MS: Ultra-High-Performance Liquid Chromatography coupled to Tandem Mass Spectrometry; UV-vis: Ultraviolet-visible spectroscopy.
